# Aggravation d'une vasculopathie polypoïdale choroïdienne après chirurgie de la cataracte

**DOI:** 10.11604/pamj.2015.21.136.7189

**Published:** 2015-06-18

**Authors:** Meriem Abdellaoui, Hicham Tahri

**Affiliations:** 1 Faculté de Médecine et de Pharmacie de Fès, Service d'Ophtalmologie, CHU Hassan II, Fès, Maroc

**Keywords:** Vasculopathie polypoïdale choroïdienne, cataracte, baisse visuelle, choroidal polypoidal vasculopathy, cataract, visual loss

## Image en medicine

Patiente de 62 ans, mélanoderme, admise pour baisse visuelle progressive de l'œil gauche. L'acuité visuelle corrigée à l'admission est de 7/10 à droite et 2/10 à gauche sans métamorphopsie. L'examen du segment antérieur montre une cataracte nucléaire bilatérale plus dense à gauche. Le fond d'œil qui est gêné par la cataracte à gauche, montre un fin remaniement pigmentaire de la macula. La chirurgie de la cataracte par phacoémulsification est réalisée à gauche sans aucun incident. Quatre jours plus tard la patiente accuse une baisse brutale de la vision à gauche avec métamorphopsie. L'examen du fond d'œil trouve un décollement séreux maculaire avec la présence de lésions profondes nodulaires orangées péripapillaires et maculaires associées à des exsudats et un décollement séreux rétinien maculaire (A et B). L'angiographie à la fluorescéine met en évidence aux temps précoces les polypes au sein du réseau vasculaire choroïdien péripapillaire et maculaire, dont l'hyperfluorescence augmente aux temps intermédiaires et tardifs (C et D). L'OCT maculaire objective un soulèvement localisé de l'épithélium pigmentaire à pente raide surmonté par le décollement séreux rétinien avec la présence d'une membrane épirétinienne (E). Le diagnostic de VPCI est retenu. Trois injections intravitréénnes de Bevacizumab (1,25mg) à un mois d'intervalle sont réalisées permettant la diminution importante du décollement séreux rétinien, une amélioration de l'acuité visuelle qui est passée de 1/10 à 7/10 corrigée ainsi que la disparition des métamorphopsies . Cependant le décollement de l'épithélium pigmentaire est resté stationnaire (F).

**Figure 1:**
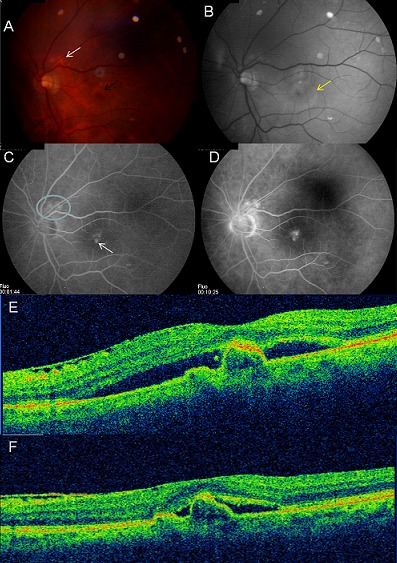
A et B: la photo couleur (A) et anérythre (B) du fond d’œil montre la présence de lésions profondes nodulaires orangées péripapillaires et maculaires (flèche noire) associées à des exsudats (flèche blanche) et un décollement séreux rétinien maculaire (flèche jaune); C et D: l'angiographie à la fluorescéine met en évidence aux temps précoces les polypes au sein du réseau vasculaire choroïdien péripapillaire et maculaire (flèche blanche), dont l'hyperfluorescence augmente aux temps intermédiaires et tardifs; E: l'OCT maculaire objective un soulèvement localisé de l’épithélium pigmentaire à pente raide surmonté par le décollement séreux rétinien avec la présence d'une membrane épirétinienne; F: l'OCT maculaire après traitement objective la diminution importante du décollement séreux rétinien avec persistance du décollement de l’épithélium pigmentaire

